# Editorial: Internal and external factors affecting polycystic ovary syndrome

**DOI:** 10.3389/fendo.2025.1594718

**Published:** 2025-04-14

**Authors:** Alexandra E. Butler, Thozhukat Sathyapalan, Harpal Randeva, Stephen L. Atkin

**Affiliations:** ^1^ Research Department, Royal College of Surgeons in Ireland Bahrain, Busaiteen, Bahrain; ^2^ Academic Endocrinology, Diabetes and Metabolism, Hull York Medical School, Hull, United Kingdom; ^3^ Department of Endocrinology, University Hospitals Coventry and Warwickshire National Health Service (NHS) Trust, Coventry, United Kingdom

**Keywords:** polycystic ovary syndrome, PCOS polycystic ovarian syndrome, genetics, lifestyle, fertility

**Figure 1:**
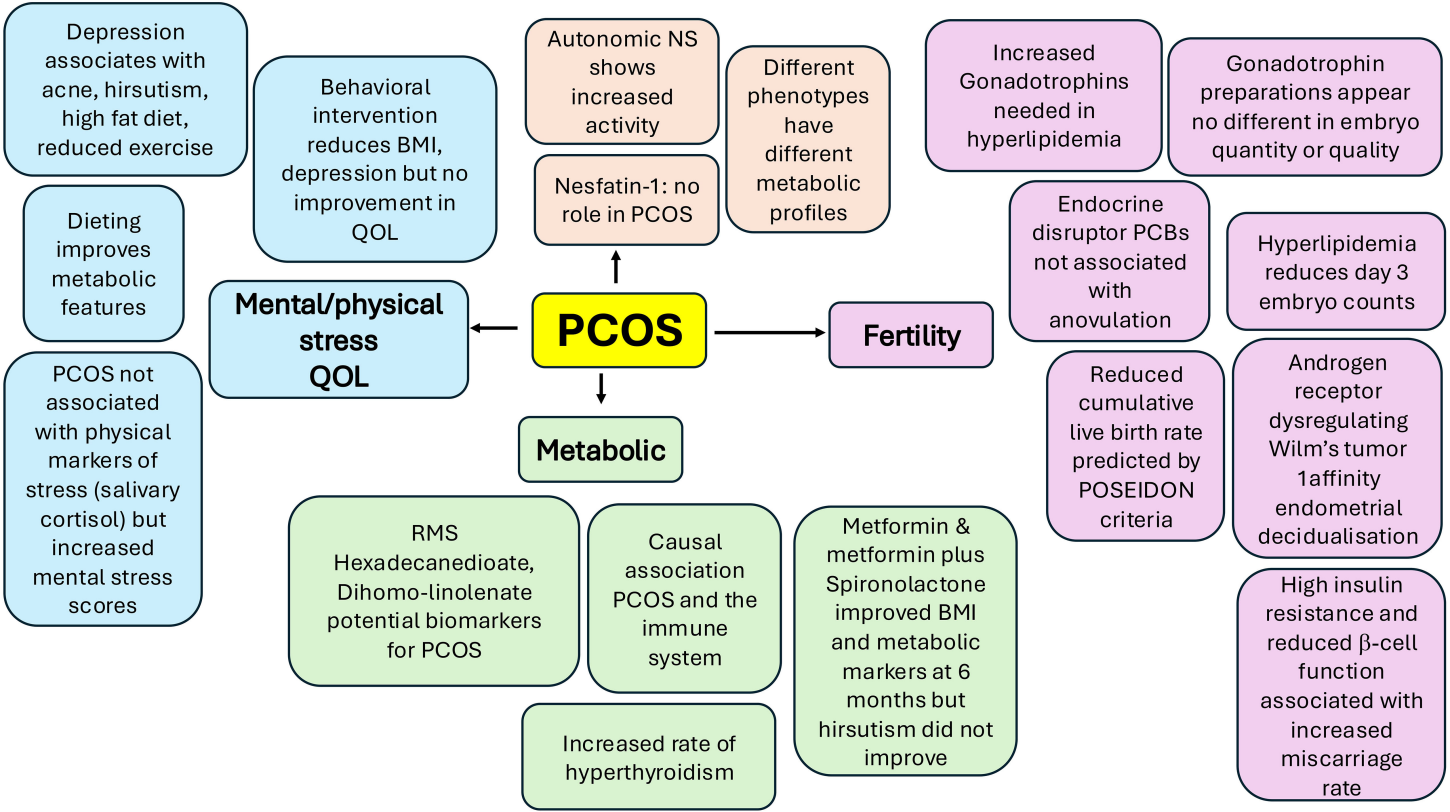
A schematic to illustrate the broad range of articles included in this Research Topic . The articles can be broadly divided into Mendelian randomisation studies (RMS), articles regarding lifestyle and PCOS pathophysiology, and fertility including in vitro fertilization studies.

Polycystic ovary syndrome (PCOS) pathophysiology is modified by a multitude of internal and external factors that go far beyond the traditional stimulators and inhibitors of endocrine response and function. This Research Topic call was to specifically include state-of-the art methodologies and review articles, with the result that 21 manuscripts were published that could be broadly divided into mendelian randomisation studies (RMS), articles regarding lifestyle and PCOS pathophysiology, and fertility including *in vitro* fertilization studies, the overview of which is shown in **Figure 1**.

Wang et al. reported a RMS showing that blood lipid metabolites and potential metabolic pathways may have a genetic association with PCOS and that there may be a causal relationship between hexadecanedioate and dihomo-linolenate and risk of PCOS. These compounds could potentially serve as metabolic biomarkers for screening PCOS and selecting drug targets; however, metabolomic studies on a robust population will be needed to answer these questions (Wang et al.) A second RMS looked at the association of PCOS and thyroid disease to address the debate as to whether PCOS is associated with hyperthyroidism, hypothyroidism, neither or both. The study reported that the occurrence of PCOS was associated with increased risk of hyperthyroidism (Zhao et al.) This suggests that, at the very least, a TSH should be measured in the investigation of PCOS; however, to confirm this, a large prospective study would likely be needed and could be combined with a long-term study looking at cardiovascular disease prevalence in PCOS. Several studies have shown a potential link between PCOS and immune system dysregulation, though the results are conflicting. In the RMS reported by Aru et al., a causal association between immune cells and susceptibility to PCOS was found suggesting a true association, but it is unclear whether the immune system is modulating PCOS or whether the converse occurs through inflammation and insulin resistance (IR) combined with obesity. It is of interest, though still unclear, whether immune modulation in PCOS is a viable treatment option particularly in the setting of infertility; the creation of new selective drugs may address this. Interestingly, a study looking at the genes for necroptosis, critical for reproductive and endocrine disorders, reported that enrichment analysis for differentially diagnostic genes for necroptosis were augmented in immune-related signaling pathways such as B cells, T cells, and natural killer cells (Wang et al.) In addition, immune microenvironment analysis revealed that differentially diagnostic genes for necroptosis were significantly correlated with 13 markedly different immune cells, data that would support the findings reported in the RMS focused upon the immune system and PCOS, as noted above (Aru et al.).

Lifestyle issues were addressed in several articles. A systematic review and meta-analysis on dietary consumption in PCOS concluded that there was limited evidence regarding the association between dairy consumption and PCOS; however, in accord with several dietary-based studies in PCOS, a low-dairy/low-starch diet may improve some anthropometric and metabolic features, likely through calorie restriction rather than any inherent food characteristics (Rastad et al.) Another study looked at the association of mild depressive states in PCOS and an unhealthy lifestyle, concluding that generally depressive symptoms in PCOS are mild and related to physical issues such as acne and hirsutism, and to poor lifestyle choices such as frequent consumption of high-fat diet, regularly staying up late and a lack of exercise, (Li et al.) all features noted to contribute to low quality of life (QOL) issues in PCOS in previous studies. This then raises the question of how this can be addressed in PCOS; a systematic review focused upon the effect of behavioral interventions on anthropometric, clinical and biochemical parameters reported that, as anticipated, behavioral intervention strategies contribute to weight loss, reduction in BMI and in waist circumference, and improvement in depression (Xie et al.) However, surprisingly, no significant improvement was observed in biochemical indices or QOL (Xie et al.), the latter being discordant with the improvement in depression reported. This data may predict that PCOS patients will not adhere to lifestyle and exercise, the cornerstone of the treatment strategy, if QOL is not improved by the intervention. The question then arises whether there is a physiological contribution to the psychological stress indices that have been reported in the literature. A study investigating both physiological and mental stress indices showed no difference in salivary cortisol levels, suggesting the physiological markers of stress are not elevated in PCOS even though the mental stress score was higher in PCOS, (Marschalek et al.) findings that are in accord with previous studies: prolactin was suggested as a measure of physical stress, though avoiding an acute stress-induced prolactin response leading to a misleading positive result may be challenging. Differing results within these reported studies for the PCOS subjects, when compared to the literature, may be a result of not defining the PCOS phenotype within the study group. This was highlighted by a report showing that differing phenotypes of PCOS were metabolically dissimilar: those with hirsutism and anovulation, and hirsutism with PCOS ovary features, had the most adverse metabolic profile including IR, dyslipidemia, impaired glucose tolerance and non-alcoholic fatty liver disease (Wen et al.).

How such research may be undertaken to identify novel research areas and knowledge gaps was reported in a bibliometric analysis for metabolic dysfunction in PCOS using software to analyze the status of the field, illustrating the key words that included lipid profile, androgen receptors, phosphorylation, luteinizing hormones, proteomics, metabolomics and gut microbiota, (Xu et al.) providing a valuable tool for researchers in the field.

In an intervention study of metformin versus spironolactone plus metformin, a not uncommon combination in clinical practice, the combination treatment improved BMI and serum androgen levels though, unexpectedly, hirsutism was not different, an outcome which differs to other published studies (Zeng et al.). After six months, metabolic indices were improved with lower IR and fasting blood glucose**;** this was, however, a relatively short duration study and it would be important to determine what happens at 12 months and beyond to see if this improvement is maintained.

A review of the autonomic nervous system (ANS) in PCOS presented evidence that there is increased ANS activation in PCOS that could be due to the induction of inflammation, for example, though whether this was a contributory cause or an effect of the underlying pathophysiology of PCOS is unclear. In addition, it is not clear whether the ANS activation contributes to the decreased QOL, emotional stress issues or depression indices or, conversely, whether these conditions cause its activation (Yu et al.)

Nesfatin-1 is a 82 amino acid peptide that is involved in glucose homeostasis, anxiety and depression and cardiovascular function and therefore would be suspected to differ in PCOS. Nesfatin-1 levels are discrepant in the PCOS literature and this question was addressed in a systematic review and meta-analysis that showed that blood nesfatin-1 levels did not differ in PCOS compared to control subjects (Wang et al.), suggesting this factor has no role in PCOS, though phenotype heterogeneity was not taken into account.

Unsurprisingly, fertility aspects accounted for nearly 50% of all accepted articles. Wilms tumor-1 is critical for endometrial decidualization and its function may be dysregulated in PCOS due to epigenetic factors allowing androgen receptors to recruit cofactors that affect the process (James et al.); this may, in part, explain the increased miscarriage rate in PCOS that is then further exacerbated by IR and β-cell dysfunction. In another report, levels of the endocrine disruptor polychlorinated biphenyl (PCB) were shown to correlate with luteal phase hormones and unexplained infertility but not in PCOS patients suggesting that PCBs are not involved and do not contribute to ovulatory dysfunction in PCOS, though they may have other independent effects on fertility.

PCOS patients, following a suboptimal response to IVF, can achieve a reasonable live birth response per fresh embryo transfer. However, the cumulative live birth rate per aspiration cycle differed in women with PCOS as defined by the Patient-Oriented Strategy Encompassing Individualized Oocyte Number (POSEIDON) criteria, indicating that prediction of future clinical outcome can be made for certain patient groups that are at risk following a poor outcome – though there was no difference for subjects with good reproductive outcomes (Jiang et al.). A further study reported that both high IR and HOMA-B, as a measure of β-cell function, were associated with an increased miscarriage rate, IR being the most important of the two (Huang et al.). This is clearly of importance in PCOS due to the higher IR that patients have, and there is a need to understand the mechanism of this increased miscarriage rate to determine if weight loss, for instance, is sufficient or if further pharmaceutical intervention for weight loss or insulin resistance is needed. A study looking at four different gonadotrophin preparations in a GnRH agonist protocol showed no difference in the preparations for PCOS for ovarian hyperstimulation syndrome or embryo quantity and quality (Hu et al.), which is reassuring for clinical practice; however, a study also reported in this Research Topic showed that the dose of the gonadotrophin in IVF may have to be increased in the presence of hyperlipidemia, and that hyperlipidemia per se may also reduce embryo quality, endometrial receptivity and clinical outcomes in PCOS patients (Yang et al.); notwithstanding, it may be difficult to address hyperlipidemia and potential treatment teratogenic effects prior to an IVF cycle. The causality of these detrimental effects of dyslipidemia were investigated in another article within this Research Topic, showing the resultant inflammation from increasing serum triglyceride and low-density lipoprotein cholesterol levels and decreasing serum high-density lipoprotein cholesterol levels, resulted in a reduction of day-3 embryo counts (Jiang et al.).

Finally, in a review article, a novel treatment for infertility was proposed using stem cells and exosomes, both of which exhibit cytokine effects that may defend against the metabolic consequences found as part of the PCOS condition (Hadidi et al.) Exosome therapy in rats has shown beneficial effect on enhancing fertility though mechanistic control of the exosome process has still not been fully elucidated. Exosomes derived from mesenchymal stem cells have been shown to suppress chronic inflammation by decreasing the generation of inflammatory mediators such as tumor necrosis factor alpha (TNF-α) and interferon gamma (IFN-γ), elevating interleukin-10 (IL-10) levels and anti-inflammatory cytokines and reducing ovarian granulosa cell apoptosis. In addition, these exosomes were shown to regulate androgen synthesis in an *in vitro* model and restore fertility in a mouse model suggesting their promise as a therapy for PCOS fertility in the future.

The diverse nature of the individual manuscripts in this Research Topic highlights the breadth of research directed towards understanding and treating the PCOS disorder. Many of the articles in this Research Topic were, in fact, complementary to others in the series providing further evidence regarding the underlying pathophysiology of PCOS and its future treatment.

